# Tracking Molecular Diffusion across Biomaterials’ Interfaces Using Stimulated Raman Scattering

**DOI:** 10.1021/acsami.2c04444

**Published:** 2022-07-08

**Authors:** Han Cui, Andrew Glidle, Jonathan M. Cooper

**Affiliations:** † Beijing Key Lab for Precision Optoelectronic Measurement Instrument and Technology, School of Optics and Photonics, Beijing Institute of Technology, Beijing 100081, China; ‡ Division of Biomedical Engineering, James Watt School of Engineering, University of Glasgow, Glasgow G12 8LT, United Kingdom

**Keywords:** biomaterial interfaces, stimulated Raman scattering, diffusion, spectroscopy, hydrogels, tissue

## Abstract

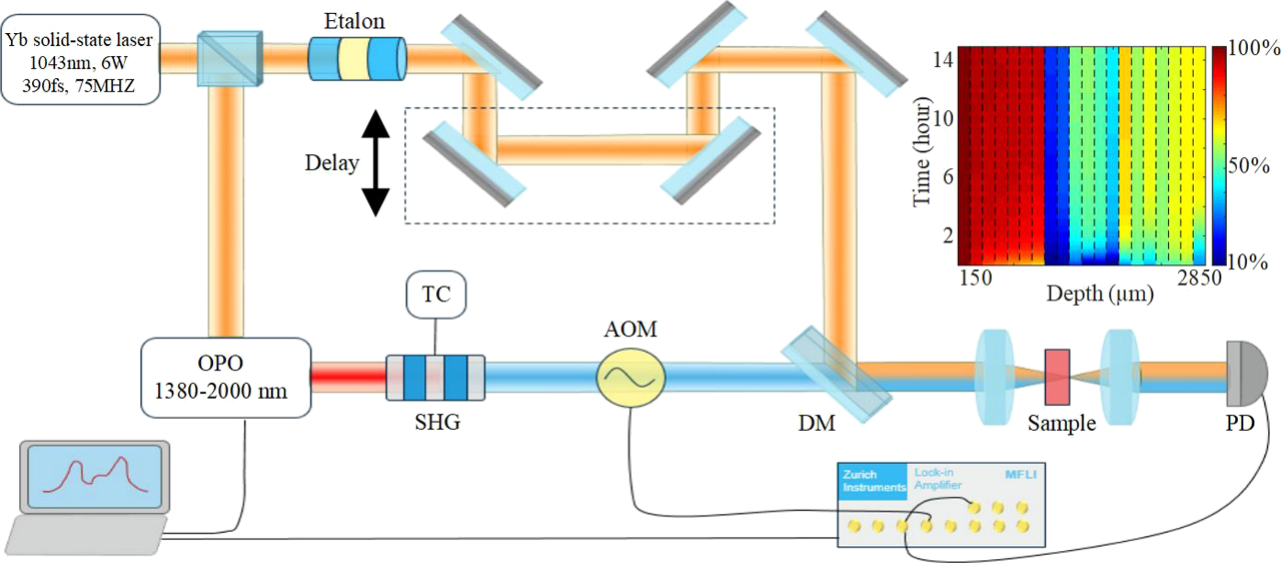

The determination of molecular diffusion across biomaterial interfaces, including those involving hydrogels and tissues remains important, underpinning the understanding of a broad range of processes including, for example, drug delivery. Current techniques using Raman spectroscopy have previously been established as a method to quantify diffusion coefficients, although when using spontaneous Raman spectroscopy, the signal can be weak and dominated by interferences such as background fluorescence (including biological autofluoresence). To overcome these issues, we demonstrate the use of the stimulated Raman scattering technique to obtain measurements in soft tissue samples that have good signal-to-noise ratios and are largely free from fluorescence interference. As a model illustration of a small metabolite/drug molecule being transported through tissue, we use deuterated (*d*^7^-) glucose and monitor the Raman C–D band in a spectroscopic region free from other Raman bands. The results show that although mass transport follows a diffusion process characterized by Fick’s laws within hydrogel matrices, more complex mechanisms appear within tissues.

## 1 Introduction

Understanding mechanisms for molecular diffusion across biological interfaces plays an important role in formulating many procedures e.g., deepening our understanding of cell signaling or drug delivery for the treatment of diseases (including for example the development of hydrogel patches for transdermal drug delivery or for insertion into wound sites to enhance healing^1^). In such cases, bioactive agents are often preloaded within hydrogels and subsequently diffuse across a biomaterial interface e.g., into tissue. Effective characterization of mass transfer, through either convective or non-convective mechanisms, provides a key predictor of the transport process kinetics and may be important in informing new biomaterials’ formulations and formats.^2^

Current methods to quantify the rates of diffusion often involve labeling, include the use of fluorescent markers with confocal imaging in a z-stack,^3^ nuclear magnetic resonance (NMR),^4^ dynamic light scattering (DLS),^5,6^ fluorescence recovery after photobleaching (FRAP),^7^ and neutron transmission.^8^ However, tagging with such labels is not always practical and may influence the molecule’s interaction with tissues or cells. In some cases where the label is large, it may also influence transport properties.

Previously, Raman spectroscopy has often been proposed as an effective method that can be used with non-labeled molecules to observe diffusion processes.^9,10^ The methodology enables quantitative analysis of a “fingerprint spectra” resulting from the characteristic molecular vibrations of the different components in a sampled volume. As a label-free and non-invasive detection technique,^9,10^ Raman microspectroscopy has emerged as a useful tool to probe molecular movement and species identification in complex samples, thereby yielding spatial concentration profiles.^11−13^

In one example, Chuchuen et al.^13^ measured the spatiotemporal concentration distribution of the antiviral drug tenofovir in a clinical gel with confocal Raman spectroscopy. Aside from biomedical applications, the technique has also been used broadly in materials science. For example, Geisler et al.^11^ performed in situ detection at the colloidal interface of a dissolving glass slide to obtain real-time spatial and temporal insights into the reaction and transport process occurring, while Peters et al.^12^ realized measurements in low-volume chip based systems, where Raman microspectroscopy combined with microfluidics was used to measure diffusion within multicomponent liquid phase systems.

Although the characteristic fingerprint of a molecule’s Raman spectrum can simplify its detection, the relative weakness of the Raman signature (with only 1 in 10^8^ photons being Raman-scattered following non-resonant, spontaneous excitation) has always proven problematic. As a consequence, fluorescence (including the autofluorescence of biological molecules) or other background scattering signals (e.g., from the molecule of interest or the surrounding matrix) can obscure a sample’s signature spectrum, hindering the application of spontaneous Raman spectroscopy.^14^

To address these problems, methods of generating or collecting Raman spectra have been developed in which these undesirable scattering features are eliminated from the measured signals. Among these, time-resolved Raman spectroscopy and stimulated or coherent techniques such as the pump-probe-based stimulated Raman scattering (SRS) spectroscopy are most notable.^15^

In the case of SRS, the Raman effect relies upon the coherence of two stimulating laser sources with a mechanism of photon-vibration energy transfer taking place, in which energy is transferred between a “pump” and a “Stokes” laser beam.^16,17^ This transfer results in the change in intensity of one or other of the beams (a change in the pump intensity is called a stimulated Raman loss or SRL, while a change in the Stokes intensity is a stimulated Raman gain or SRG). Typically, the method of measuring the Raman signals simply involves monitoring the SRL or SRG as a photocurrent generated in a simple silicon diode.

Apart from the advantages of being free from fluorescent and non-resonant backgrounds,^18^ SRS also amplifies Raman signals by several orders of magnitude due to the coherent excitation of molecular vibrations with the ultrashort laser pulses, enabling real-time chemical imaging of non-labeled species. Together, these advantages overcome many of the problems associated with the complex sample matrices found in biomedical applications.^19−23^ For example, Chiu et al.^19^ explored the diffusion of pure solvents into the dense keratinous material of human fingernails, showing that this penetration was both related to the molecular size and limited to tens of micrometers even after 24 h for the larger solvent molecules. Dong et al.^20^ used polarization-sensitive stimulated Raman scattering from fingerprint C=C stretching vibration to visualize amphotericin B (AmB) in single fungal cells, while Ji et al.^21^ applied SRS microscopy to image amyloid plaques, one of the key pathological features of Alzheimer’s disease (AD), in the brain tissue of an AD mouse model (demonstrating SRS as a rapid, label-free tool to differentiate misfolded from native proteins).

In the work below, we now describe the use of stimulated Raman scattering to systematically investigate the diffusion behavior of deuterated glucose (as a readily available model small molecule) in a hydrogel/tissue structure to mimic the drug delivery process. In contrast to the diffusion studies above, in our paper, we explore how the technique could be used to probe the mass transport of small molecules in a carrier solvent (where the concentrations of active species are lower) into hydrated and porous material. To illustrate the motivation for using stimulated (rather than spontaneous) Raman scattering in studies that involve naturally occurring biomaterials (e.g., tissue), Supplementary Figure S1 shows the difficulty of resolving spontaneous Raman peaks when a high, broad-band, scattering background is present: Supplementary Figure S1 shows that although the C–D peak of deuterated glucose can be seen clearly in samples with low background scattering (such as transparent hydrogels), when the glucose is imbibed into a highly scattering sample such as a tissue slice, the C–D peak is significantly harder to discern from other background and fluorescence scattering features, even when the glucose concentration is very high (e.g., 1 M).

A further advantage of the SRS methodology is that a lock-in detection method can be used to modulate the intensity of the pump laser source, significantly improving the sensitivity. As a consequence, for example, detection of the C–D band of deuterated glucose gives a signal that is easily distinguished from the background scattering signals of, for example, the tissue components of a sample. Importantly too, in SRS measurements, it has been shown that the signal detected is proportional to the concentration of the species being probed^24^ (see also Supplementary Figure S2). This, coupled with the rapid readout times from the silicon detector, means that the technique is highly suited to tracing and visualizing, for example, glucose diffusion in real time. This allows data to be collected from highly scattering samples with good temporal resolution, enabling the determination of whether the observed mass transport follows the classical Fick’s law mechanism or other models and, where appropriate, extracting the corresponding diffusion coefficients.

## 2 Methods and Materials

### 2.1 Stimulated Raman Scattering System

**Figure 1:**
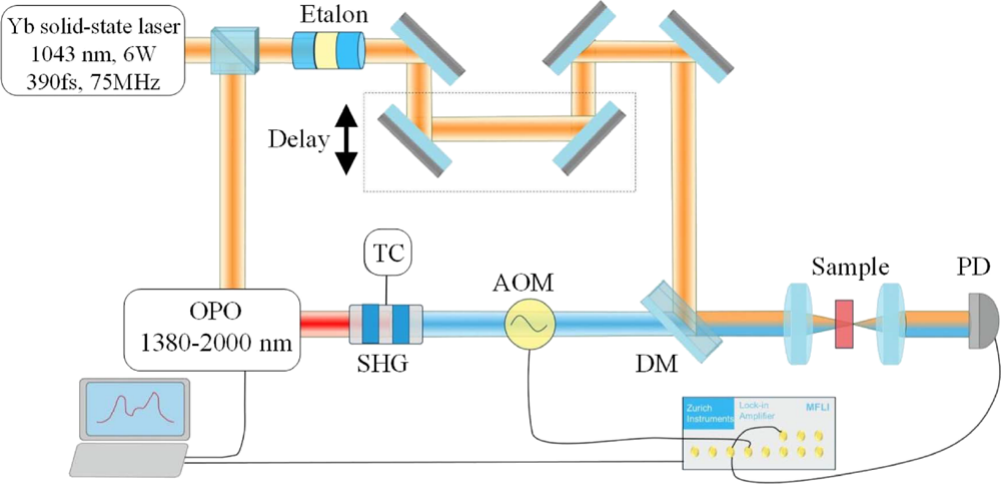
Schematic experimental setup. An Yb solid-state oscillator allows synchronous generation of the Raman Stokes and pump beams. Using an etalon, the narrowband Stokes beam with picosecond pulse duration is obtained, while the Raman pump beam is generated by pumping an optical parametric oscillator (OPO), which is subsequently frequency-doubled in a periodically poled crystal employing the effect of spectral compression. SHG: second-harmonic generation, DM: dichroic mirror.

The stimulated Raman Scattering (SRS) system shown as a schematic in Figure 1 is based upon an Yb solid-state oscillator with 1043 nm central wavelength as the excitation source. A proportion of the light used as the Stokes beam passes through the beamsplitter, etalon, and delay system, while the remainder is reflected by the beamsplitter to enter the fiber-feedback optical parametric oscillator (OPO) (Stuttgart Instruments, Germany), which is subsequently frequency-doubled using a periodically poled lithium niobate crystal to give second-harmonic generation (SHG) as the pump beam. The Stokes beam and pump beam are converged by a notch filter and then focused on the sample by a lens, with the Raman signal collected by a photodiode placed on the other side of the sample as described in our previous paper.^25^

In the current SRS system constructed here, a pulse laser based on an Yb solid-state oscillator (M-FEMTO-LAB-Yb, Montfort Laser, Austria) was used as the excitation source, which provides 6 W of average output power at a 1043 nm central wavelength with ca. 390 fs pulses and a 75 MHz pulse repetition rate. A 50× objective (0.55 NA Leica, Germany) was used as the focus objective, and a 40× objective (0.55 NA, Zeiss, Germany) was used as the collection lens. The photodiode (PDA100A2, Thorlabs, USA) sensor was used as the signal detector, and a lock-in amplifier (MFLI, Zurich instruments, Switzerland) works as both modulation tool for the pump signal and for weak signal amplification to pick up the detected SRS signal.

The use of these objectives, with numerical apertures of ∼0.55 result in a focused spot of the order of 4 μm in diameter as verified by monitoring the signal intensity due to the C–H band in a hydrogel while scanning the 40× collection objective in directions perpendicular to the laser beams using motorized stages. Similarly, the thickness of the sample voxel being probed in the SRS measurement was estimated by translating the collecting objective along the axis of the laser beams; this indicated that the length of voxel was ∼20 μm. i.e., the SRS system probes voxels of ∼4 μm × 4 μm × 20 μm.

### 2.2 Sample Holder

**Figure 2:**
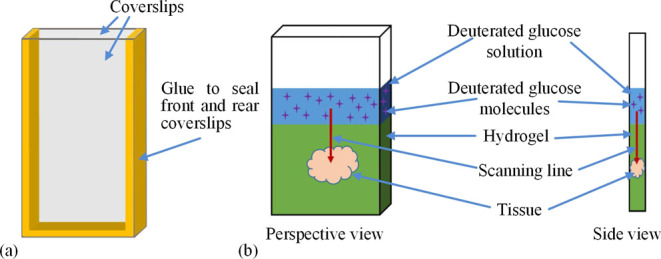
Sample holder and tissue embedded in hydrogel: (a) Thin-layer sample holder formed by adhering two coverslips together on three sides; (b) arrangement of the tissue and hydrogel samples contained within the thin-layer sample holder covered with a deuterated glucose solution (purple crosses denote glucose molecules); an exaggerated perspective is shown for clarity, and the side view illustrates how the tissue sample is tightly sandwiched between the two coverslips. The red line indicates the path of the SRS laser beams when the sample is scanned to probe the glucose concentration at different depths from the solution interface, i.e., it is midway in both the lateral (side-side) direction and in the sample thickness direction so as to minimize edge effects.

In order to measure the transport of glucose into hydrogel and tissue samples, a glucose solution was added to the top of a thin layer cuvette constructed from glass coverslips that contained the hydrogel or tissue slice (Figure 2).

The sample holders used in this study (Figure 2) were bespoke cuvettes comprising two #1.5 coverslips spaced apart by using either a third coverslip (of ∼0.2 or ∼0.4 mm thickness) or a microtomed tissue slice to separate them during the adhesive process required to make a cuvette that was sealed on three sides. The internal path length of the cell and the outer coverslip thicknesses were accurately determined using a micrometer. The direction of the SRS lasers were perpendicular to the front face shown in the perspective views of Figure 2, with the detector placed behind the cuvette as in a transmission measurement configuration. In addition to the tissue in hydrogel sample shown in Figure 2, samples for individual hydrogel or tissue measurements were prepared in a similar fashion.

For those cases involving the study of hydrogels, the sample was polymerized within the cuvettes by exposure of the pre-polymer solution to UV light (see below for details). The three different types of sample comprised either a polymer-based hydrogel, a microtomed avian (chicken) breast muscle model, or a sample in which the avian tissue was embedded in the hydrogel (with the hydrogel only being in contact with the edge of the tissue slice; Figure 2).

Once prepared and the cuvette’s assembly and adhesion was complete, samples were filled with 0.2 M phosphate-buffered saline (PBS) and left overnight to elute any unreacted monomer species and ensure the tissue slices were fully hydrated prior to the experiments involving per-deuterated glucose (d-glucose-1,2,3,4,5,6,6-*d*_7_, 552003, Sigma-Aldrich, Germany) solutions.

Note that by using the above method of cuvette assembly, in which the tissue is sandwiched between two glass coverslips, there could be concerns that when the test glucose solutions are added to the cuvette from above the tissue, the solution could simply run down a microscopic gap at the interface between the glass and tissue. This would provide a rapid transport path to parts of the tissue that were far from the bulk of the glucose solution. Thus, to prevent this from happening, the tissue sample is held in the sandwich using the pressure of firmly wound micrometer anvils during the gluing process.

Nevertheless, to ensure that the concentrations measured by the SRS probe were not influenced by “edge” effects at the coverslip interfaces, the cuvette was translated along the axis of the laser beam while monitoring the signal from the O–H band of water. This signal was present in the tissue as well as the hydrogel and glucose solution but not in the glass coverslips. The signal, which was effectively zero when the laser beam focus was in either of the two coverslip glass walls, rose to a maximum when the focus was half way into the center of the ∼200 μm thick samples, also served to confirm the absence of a thin layer of water between the coverslips and tissue or hydrogel material. The subsequent C–D measurements were taken at the translation position corresponding to the maximum O–H signal.

### 2.3 Diffusion Measurements and Data Acquisition

As indicated above, the uniquely assignable C–D band of per-deuterated glucose provides a distinct Raman marker; nevertheless, in order that data acquisitions in which small changes in glucose concentration could be detected with short data acquisition times (10–100 ms), a high concentration of glucose was used.

Fluorescein was added to the glucose solution and served, from a pragmatic point of view, to be a convenient visible marker to monitor the progress of the glucose mass transport (since it is of a similar size) and thereby authenticate the deuterated glucose concentration profiles obtained from the SRS measurements in these model experiments.

Transmission SRS measurements were acquired by passing the laser beams through the cuvette mounted in a motorized vertical translation stage. A Labview program controlled the stage movement. This program cyclically scanned the cuvette up and down in a series of 150 μm steps (e.g., along the red line shown in Figure 2b), pausing after each 150 μm movement to acquire the SRS signal. Typically, 17–25 150 μm height translation steps were used in a single top-to-bottom scan to probe the magnitude of the C–D band in each of the solution above the hydrogel or tissue sample and at different distances from the solution/sample interface into the sample (effectively probing the glucose concentrations at different lateral depths into the sample).

The start of the height (*z*) scan started with the laser beams passing through the deuterated glucose solution at a height of around 1 mm above the hydrogel (or tissue) interface and finishing around 2.5 mm below the interface. At each height position, a series of 10–100 ms SRS signal acquisitions were made, and these were collected for 15 s to monitor any transient changes in response. Typically, this resulted in a top-to-bottom scanning time of ∼400 s and thus determined the temporal resolution of any diffusion or kinetic data (note that smaller numbers of height steps and shorter SRS signal accumulation times could have been used if the processes occurring were found to be faster).

The top-to-bottom height scanning was cycled for around 15 h to quantify the diffusion processes over an extended period. During this period, the SRS signal was collected for the C–D band at 2100 cm^–1^. Data were also being collected at 1990, 2260, 2520, 2950, and 3390 cm^–1^ both before and after the 15 h scanning period. These spectral regions corresponded to the “background” regions at either side of the C–D band, the C–H band of the hydrogel or tissue, and the O–H water band, respectively. Importantly, collection of this data was used in the data processing routines to verify that the changes in C–D band signal were due to the ingress of C–D species and not to changes in the sample background/instrument signal levels. Furthermore, they also served to monitor any changes in the water and polymer concentrations that might arise as the glucose diffused into the hydrogel or tissue sample and occupied void space in these matrices.

### 2.4 3D PEG Hydrogel Scaffold Fabrication

Polyethylene glycol (PEG) hydrogels were prepared by mixing a photoinitiator (Irgacure 2959, Sigma-Aldrich) with a 4-Arm PEG-Acrylate (molecular weight: 10 kDa, Creative PEGWorks) solution. Hydrogel scaffolds were manufactured by photopolymerization of a solution comprising concentrations of 10% (w/v) PEG and 0.05% w/v Irgacure 2959 in phosphate-buffered saline (PBS, Sigma-Aldrich). The mixed solution was poured into a sample holder for irradiation with UV light (320–390 nm, 5 mW/cm^2^ with 200 s exposure to achieve the polymerization throughout the gel).

### 2.5 Data Processing

For the purposes of data analysis, the “*t* = 0” time of each experiment was defined by the moment when the mixture of deuterated 2 M glucose and 0.1% fluorescein solution was added to the cuvette to form a solution layer above the gel or surrounding the tissue (Figure 2). Data processing was implemented with Matlab (version 2018b, MathWorks, USA) in order to extract the data corresponding to the discrete height steps from a long time series. The sample scanning process was controlled and synchronized with data acquisition by an in-house written Labview program. As indicated above, the C–D band signal was corrected for the background signal from the SRS system and the sample cuvette by subtracting the signals at 1990 and 2260 cm^–1^ at either side of the C–D peak (typically, these background levels were only a few percent of the C–D signal), again using Matlab routines.

## 3 Results and Discussion

### 3.1 Diffusion Process in the Hydrogel

The PEG hydrogels used provide hydrophilic polymer networks in many biomedical engineering studies often have a water content of over 50% v/v and consequently have been studied for applications in several fields including drug delivery, with one of the key features being the capacity for the mass transport of small molecules within these gels. Here, to explore the dynamics of this process, we used tracking of the transmission Raman signal corresponding to the C–D band of per-deuterated glucose as the molecules move through the polymer network of the hydrogel.

**Figure 3:**
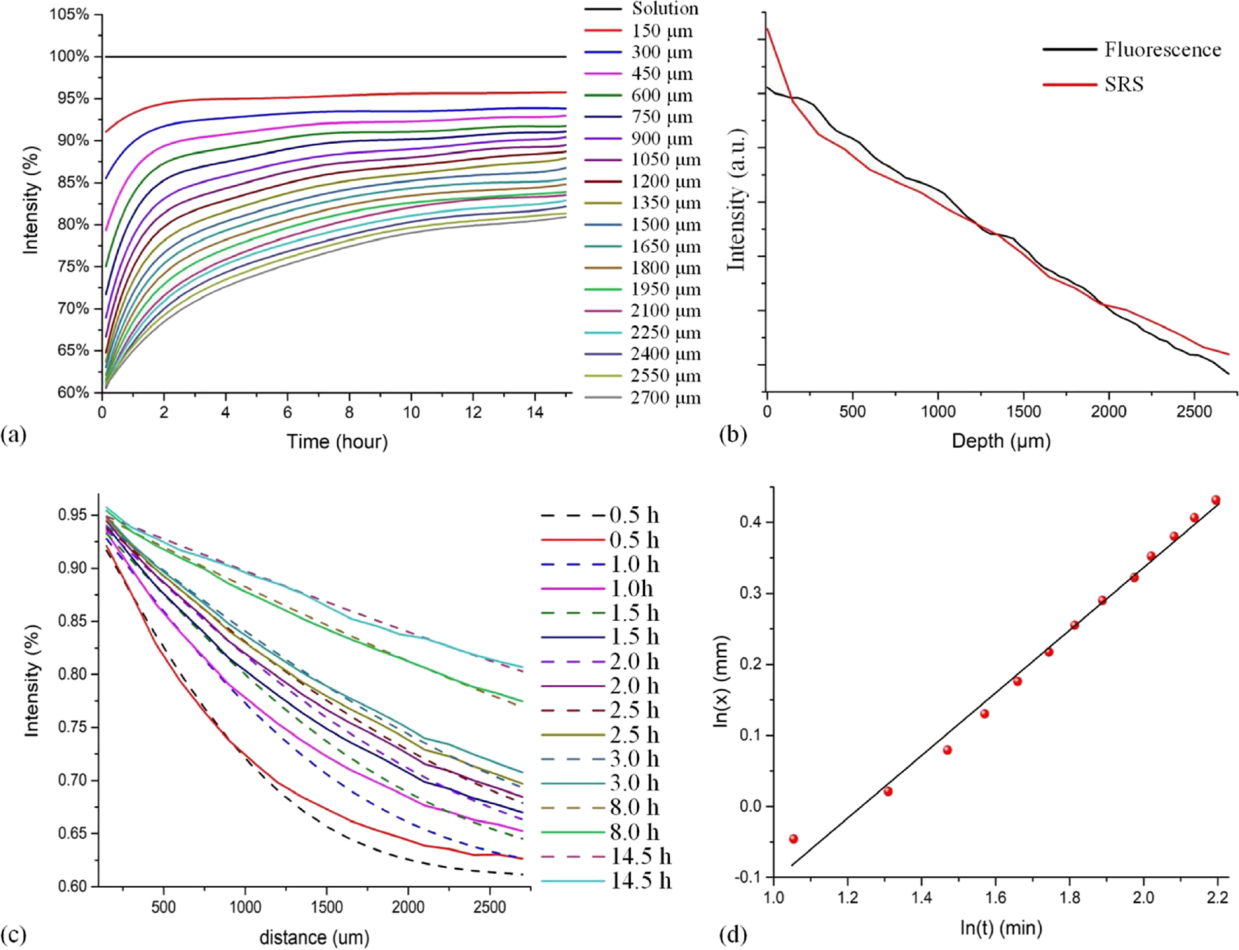
Results of deuterated glucose particle diffusion in hydrogel: (a) Diffusion profile of deuterated glucose molecules at all steps down to 2.7 mm below the interface in pure hydrogel sample as measured by SRS signal. (b) Comparison of SRS signal from deuterated glucose (red line) and fluorescence (black line) 15 h after addition of the combined glucose/fluorescein solution. (c) Variation of measured C–D band intensity with depth into the sample for selected times after addition of the glucose/fluorescein solution, along with variations predicted by a Fickian model using diffusion coefficients calculated in Table 1 (dotted lines). (d) Log–log plot of the diffusion depth (*y* axis) vs time for depths at which the glucose concentration has reached 70% of that found in the bulk solution above the hydrogel sample (the *x* axis time data is calculated from the time taken for the concentrations at these points to reach 70% of that found in the solution phase).

Signals were collected while scanning the sample vertically over a distance of several mm and over a period of ∼15 h to quantify the evolution of concentration profiles over a range of distances from the solution/sample interface (Figure 3a). Here, the top black line corresponds to the relative SRS Raman intensity of the C–D band in the aqueous layer above the hydrogel (designated as 100% intensity). The colored lines show that the C–D band intensity within the gel is lower than in the solution, reflecting the lower concentrations of glucose within the gel (albeit that they continuously increase with time as mass transport progresses).

In common with diffusion processes described by Fick’s laws, mass transport is fast in the early stages at all depths (bearing in mind that in the absence of glucose, the relative signal at *t* = 0 is 0%) and after longer periods of immersion in the glucose solution, the intensity plateaus corresponding to the glucose concentration in the gel reaching a steady state. As indicated earlier, in these exploratory experiments, to check that the C–D band SRS Raman measurements tracked the glucose concentration accurately, we added a low concentration of fluorescein to the glucose solution so that this could be imaged with a gel reader (Syngene PXi Touch) at the end of the experiments. Figure 3b shows that the fluorescence intensity and C–D Raman intensity track each other well when the measurements from the different instruments are suitably scaled, i.e., the measurements of the visible fluorescein marker support the proposition that the invisible glucose molecules have also penetrated deep into the tissue.

Figure 3a also shows that for depths up to ∼500 μm, the plateau of C–D intensity corresponds to 90–95% of that for the solution layer above the gel, indicating that this uppermost layer of the gel is very highly hydrated. However, at deeper positions into the gel, the C–D intensity (or glucose concentration) only reaches 80% of the solution value even after a period of 15 h. The lower values attained deeper into the gel could be due to the slowness of the diffusion process or a difference in the hydrogel polymer density at these depths. Such a difference in density could manifest itself in both a change in the glucose diffusion coefficient and the loading capacity of the gel. To explore and better understand these possibilities, we examined how well the glucose mass transport data fit to Fick’s descriptions of diffusion.

If we assume that the mass transport process in the hydrogel is a non-steady-state diffusion step, this process can be described by Fick’s second law, as given in eq 1:
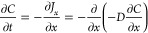
1Here, *C* is the concentration, *t* is the time, *D* is the diffusion coefficient, and *x* is the diffusion depth.

When the diffusion coefficient *D* is constant, eq 1 can be simplified to eq 2.
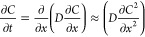
2

The solution for eq 2 is shown as eq 3.
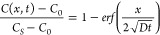
3Here, *C_o_* is the concentration of the moving species in the bulk aqueous phase, *C_s_* is the initial concentration in the hydrogel, *C*(*x*, *t*) is the concentration at time *t* and distance *x* from the interface, and *erf* is the error function, with the following definition as eq 4.
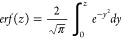
4

Based on these equations, we calculated an estimated diffusion coefficient for a number of different times using the relative glucose concentrations at different depths. The corresponding calculated diffusion coefficients are shown in Table 1. With these estimated values, predicted concentrations (expressed as relative C–D intensity values) at different depths and after different durations are plotted as the dashed line in Figure 3c.

**Table 1:** Estimated Diffusion Coefficients with Time

time (h)	0.5	1.0	1.5	2.0	2.5	3.0	8.0	14.5
estimated diffusion coefficient ×10^–6^ (cm^2^/s)	3	2.5	2.5	2.5	2.5	2.5	2.3	2

The comparison between the measured diffusion process lines (the solid line) and the predicted lines (the dash line) of Figure 3c was good for all combinations of depth and time except for deep depths at early times. Here, the measured data suggest that the glucose concentration is higher at deeper depths than would be the case based on the average diffusion coefficient estimated for the early time measurements. This higher value could indicate that the diffusion is faster at depths further away from the polymerized gel’s interface, possibly corresponding to a difference in the degree of crosslinking cf. the formation of a “skin-like” gel structure at the air/polymer interface during the photopolymerization process.

How well the concentration–depth–time data fit Fick’s diffusion laws can be confirmed by plotting the combinations of distance and time that result in a particular concentration of glucose within the gel, viz., following eq 3, the time-distance pairs that correspond to a certain value for *C*(*x*,*t*), the right hand side of eq 5 should be a constant:
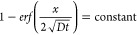
5

Consequently, it follows from the requirement for eq 5 to be a constant that diffusion depth is proportional to the square root of time, as in eq 6.
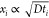
6

To examine whether the data fit this model, as an example, Figure 3d records combinations of time and diffusion depth for which the concentration of glucose is 70% of the bulk value. When plotted as a log–log plot and considering all the data in the plot, the slope of the best fit line is 0.44, while the theory predicts that it should be 0.5. This lower value is attributable to the diffusion distance estimated for the first few time points, and if these points are excluded, the gradient increases to 0.48.

Aside from the possibility that the mass transport is not purely described by Fick’s assumptions, the removal of the first few time points leading to an increase in the above gradient could reflect that either the *t* = 0 time is inaccurate or a small amount of mixing occurs between the glucose solution and a thin water layer on top of the gel when the glucose solution is added to the cuvette (leading to a small, temporary, reduction in the glucose concentration).

In summary, although the C–D Raman band SRS data for hydrogel samples do not fit the Fick diffusion model perfectly, the agreement is sufficiently close to suggest that, if the material is close to homogeneous, SRS measurements are a reliable way to track the movement of untagged molecular species.

### 3.2 Diffusion Process in Tissue

**Figure 4:**
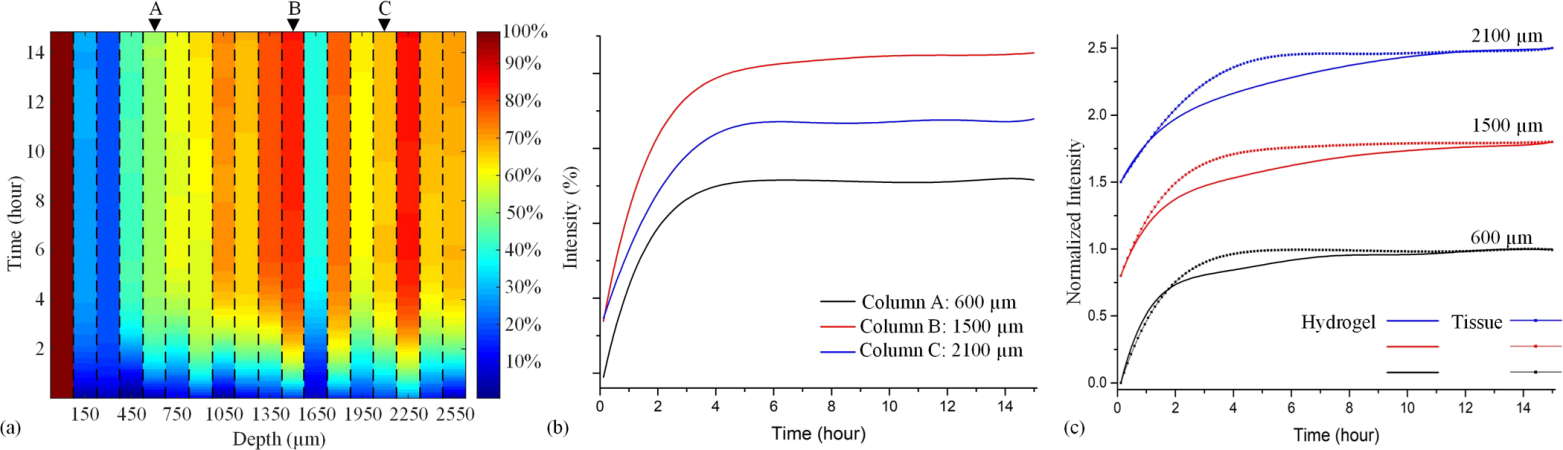
Results of deuterated glucose particle diffusion in tissue: (a) Diffusion profile of deuterated glucose molecules at height steps spaced 150 μm steps starting just above the tissue/solution interface and progressing down to 2.55 mm below the interface in tissue sample surrounded by glucose solution (cf. Figure 2 without the hydrogel). (b) Evolution of the glucose concentration at depths corresponding to positions A, B, and C of (a). (c) Comparison of the glucose concentrations at selected sample depths in hydrogel and tissue samples. Note that the curves in (c) are offset for clarity of presentation, with the plateaus at *t* = 15 h having a normalized intensity of 1.0.

Since an aim of many drug delivery strategies using hydrogels is to be able to deliver the active molecules deep into the subcutaneous tissue, we also explored the mass transport process within and at the interface of microtomed muscle tissue slices. Thus, Figure 4 shows the results of measurements by SRS monitoring of the C–D band, with Figure 4a showing the movement of glucose from solution to tissue in a heat map format.

As before, the glucose concentration was measured at different depths into the sample up to distances of ∼2.55 mm from the tissue/solution interface. Whereas for the hydrogel, the glucose concentration decreases monotonically with depth for a given time (and increases with time for a given depth; Figure 3), Figure 4a shows that as the depth being probed increases from step to step, there is some variability in the concentrations, indicating that any attempt to analyze the data in terms of simple single Fick diffusion model over the macro scale could be misleading.

Instead, if the sample is considered as a non-homogeneous structure in which dense regions of tissue are interspersed in a network of conducting channels, it is possible to envisage that for some parts of the tissue slice, glucose would be transported there more rapidly than if it had only to traverse the dense regions of tissue that were between the particular point being measured and the physically closest part of the tissue/solution interface. Indeed, the observation that for this particular sample, there is a region of ∼300 μm at the solution/tissue interface that is devoid of glucose (the 150 and 300 μm slices) and yet the glucose clearly penetrates into much deeper depths within the sample indicates the presence of multiple transport routes. Similarly, there is a second region of the sample deeper in the slice (∼1650 μm) into which no glucose penetrates yet beyond which there are regions of significant glucose concentrations. As would be expected for inhomogeneous slices, it was found that the presence and positions of regions of low glucose concentration varied from sample to sample. This could, in future be probed in greater detail through multidimensional (*xyz*) SRS mapping. Notwithstanding this, it is notable that for a given position within the tissue, the glucose concentration always increased monotonically with time, indicating that whatever mass transport routes are in operation for particular regions of the tissue, they do not change during the course of these measurements. This is further illustrated in Figure 4b, which shows the time course of the glucose profile for the depths indicated by labels A, B, and C in Figure 4a.

A further insight into the differences in internal mass transport networks of the hydrogel and tissue samples is obtained by comparing the rate of mass transport in the tissue with that in the hydrogel (Figure 4c). Here, the concentration–time data for the two systems at similar distances from the solution interface are overlaid. From this data, it can be seen that the mass transport in the tissue sample is faster than in the hydrogel, suggesting that regions of the tissue are more porous than the hydrogel on a macroscopic scale even though the hydrogel is very hydrated (80–90% water based on the polymerization conditions).

### 3.3 Diffusion Process in Tissue Embedded in Hydrogel

**Figure 5:**
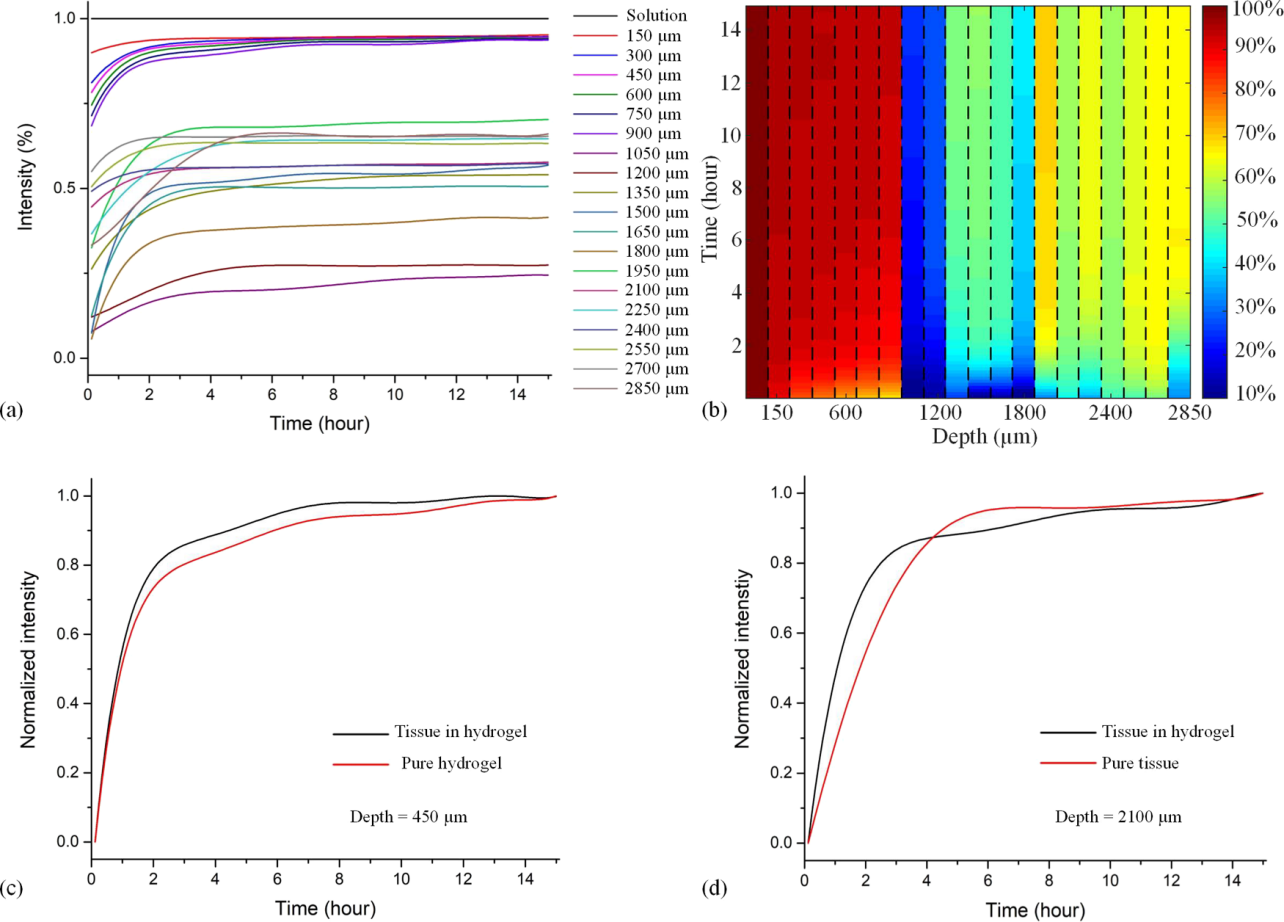
Results of deuterated glucose particle diffusion in sandwich sample. (a) Diffusion profile of deuterated glucose molecule at all the steps down to 2.85 μm below the interface in tissue embedded in hydrogel sample by SRS. (b) 2D view of (a). (c) Comparison of the diffusion profile at a distance of 450 μm below the solution/hydrogel interface or the hydrogel/tissue interface for the pure hydrogel and the tissue in hydrogel samples, respectively. (d) Comparison of the diffusion profile in the tissue at 2100 μm below the hydrogel/tissue or solution/tissue interface in the tissue in hydrogel or pure tissue in glucose solution samples. Note that these two depths were chosen as being relatively close and far from the source of the *d*^7^-glucose, i.e., the solution or hydrogel phase.

Finally, to mimic a biomedical application, we examined a third example consisting of tissue embedded into the hydrogel (Figure 2), in which the deuterated glucose solution is added to the top of the hydrogel layer. As before, 19 depth steps were performed, this time focusing on traversing the hydrogel/tissue interface, with 6 × 150 μm steps being in the hydrogel. A representative spatiotemporal distribution map of the concentration found for hydrogel/tissue samples is shown in Figure 5a. The most noticeable difference between the profiles for the hydrogel and tissue parts of the sample is that at long times, the concentration of glucose within the tissue appears to be lower than in the hydrogel. This may reflect the lower water content of the tissue, restricting the amount of glucose that can be taken up.

If the time course plots for mass transport into the gel and into the tissue are separately normalized to their long-exposure time (15 h) values, as in Figure 5c,d, it can be seen that the relative rate of increase in glucose concentration is generally faster in the tissue slice compared to the hydrogel layer, as expected from the preceding results.

## 4 Conclusions

In this paper, we demonstrate that stimulated Raman scattering is an effective way for measuring transport properties of molecules in thick and highly scattering tissue samples. Unlike spontaneous Raman and many other traditional methods, this approach is neither subject to fluorescence interference nor reliant on labeling samples with large “tags”. Instead, the method exploits Raman spectroscopy’s feature of being a non-invasive method for identifying and quantifying molecules in a mixed environment. For the simple model systems studied here, the proposed method provides fundamental insights into the mass transport processes occurring, illustrating that such SRS-based methods offer future promise in the study of more complex systems. We also believe that in the future, this technique opens up the possibility of using more sophisticated fingerprint identifications to probe evolving concentration profiles of macromolecular structures within tissue samples in vivo. The technique also has the potential to demonstrate ligand binding where a molecule may bind to particular features in the tissue (at which point diffusion would stop), including for example antibody binding to cells.

## Supporting Information Available

Spontaneous Raman spectra of deuterated (*d*_7_-)glucose in different samples; calibration curve of SRS signal and deuterated (*d*_7_-)glucose concentration (PDF)
