# Supplementary material for: Tracking Molecular Diffusion across Biomaterials’ Interfaces Using Stimulated Raman Scattering
Source: ACS Appl Mater Interfaces. 2022 Jul 8;14(28):31586–93. doi: 10.1021/acsami.2c04444 (PMC9305705; doi:10.1021/acsami.2c04444)
Supplement: Supplementary file 1 — am2c04444_si_001.pdf [file am2c04444_si_001.pdf]

Supporting Information

Tracking Molecular Diffusion across Biomaterials' Interfaces using Stimulated Raman Scattering

Han Cui,[1,2] Andrew Glidle,[2] Jonathan M. Cooper [2]*

1. Beijing Key Lab for Precision Optoelectronic Measurement Instrument and Technology, School of Optics and Photonics, Beijing Institute of Technology, Beijing 100081, China

2. Division of Biomedical Engineering, James Watt School of Engineering, University of Glasgow, Glasgow, G12 8LT, United Kingdom

* E-mail: Jon.Cooper@glasgow.ac.uk

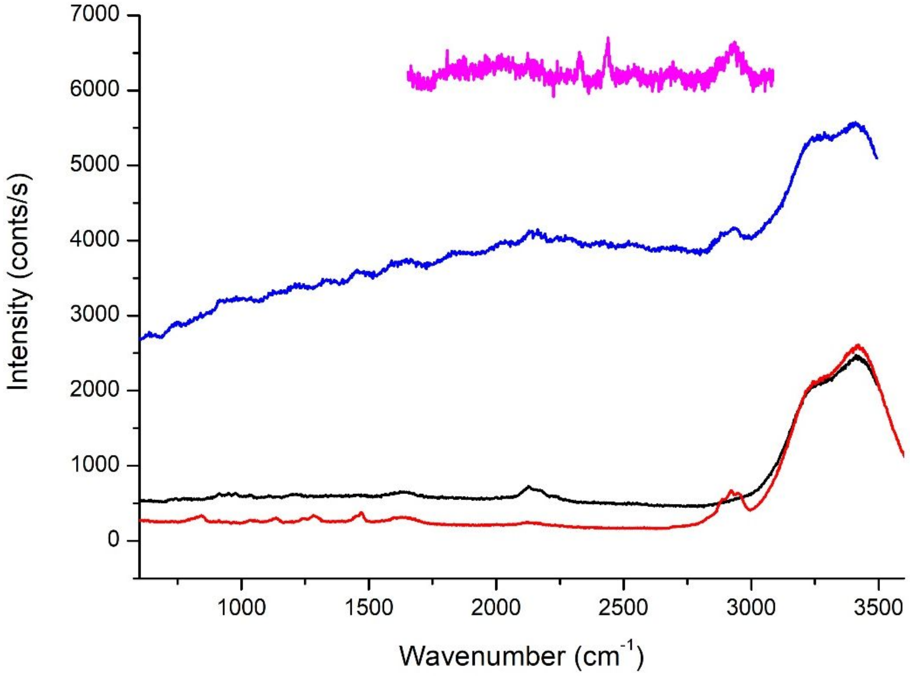

Figure S1. Comparison of spontaneous Raman spectra collected on a Jobin Yvon HR spectrometer. Line in magenta is the Raman spectra of 1 M d^7-glucose in chicken breast slice, line in blue is the Raman spectra of 0.1 M d^7-glucose in chicken breast slice, line in black is the Raman spectra of 0.1 M d^7-glucose in water, line in red is the Raman spectra of 0.1 M d^7-glucose in hydrogel. Spectra collected using a 63x, 0.75 NA objective on an Olympus IX microscope. Tissue and hydrogel samples were held in similar cuvettes to those used for the SRS experiments described in the main text. Acquisition times were 10 – 100 s, laser power 20 mW, 532 nm.

Note: Peaks at 2300 cm^{-1} and 2450 cm^{-1} in the 1 M glucose sample are from the Raman spectrometer/detector; the peak at 2850~2900 cm^{-1} in the hydrogel and tissue samples corresponds to C-H band of the organic (protein or hydrogel) matrix. The C-D peak at 2150 cm^{-1} is only clearly discernible in the solution and hydrogel spectra.

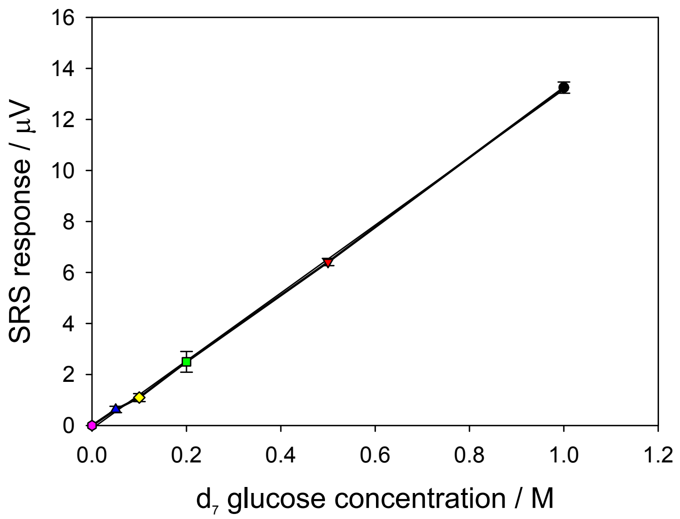

Figure S2. SRS response for d7-glucose concentrations measured in 1 mm pathlength cuvette with 50x and 40x objectives; average of triplicate measurements; $r^2 = 0.999$.
